# Role of age, sex, and specific provoking factors on the distal versus proximal presentation of first symptomatic deep vein thrombosis: analysis of the SWIss Venous ThromboEmbolism Registry (SWIVTER)

**DOI:** 10.1007/s11739-021-02878-7

**Published:** 2021-11-03

**Authors:** David Spirk, Tim Sebastian, Jürg Hans Beer, Lucia Mazzolai, Drahomir Aujesky, Daniel Hayoz, Rolf Peter Engelberger, Wolfgang Korte, Nils Kucher, Stefano Barco

**Affiliations:** 1 Institute of Pharmacology, University of Bern, Bern, Switzerland; 2 Department of Angiology, University Hospital Zurich, Raemistrasse 100, RAE C 13, 8091 Zurich, Switzerland; 3 Department of Internal Medicine, Cantonal Hospital Baden, Baden, Switzerland; 4 Clinic of Angiology, University Hospital Lausanne, Lausanne, Switzerland; 5 Division of General Internal Medicine, Bern University Hospital and University of Bern, Bern, Switzerland; 6 Department of Internal Medicine and Division of Angiology, Cantonal Hospital Fribourg, Fribourg, Switzerland; 7 Department of Internal Medicine, Cantonal Hospital St. Gallen, St. Gallen, Switzerland; 8 Center for Thrombosis and Hemostasis, University Medical Center Mainz, Mainz, Germany

**Keywords:** Age, Sex, Isolated distal deep-vein thrombosis, Provoking risk factors

## Abstract

We aimed to evaluate the impact of age, sex, and their interactions with provoking risk factors for deep vein thrombosis (DVT). In addition, we intended to provide additional insights on risk factors associated with the isolated distal versus proximal presentation of first symptomatic acute DVT, both being characterized by different prognosis. In the present analysis from the SWIss Venous ThromboEmbolism Registry (SWIVTER), we compared demographic and baseline characteristics in patients with isolated distal (*n* = 184; 35%) versus proximal (*n* = 346) DVT of the lower limbs without symptomatic pulmonary embolism, and identified factors related with the presenting thrombosis location. In the overall population, mean age was 59 ± 19 years, 266 (50%) were women, 106 (20%) patients had cancer, 86 (16%) recent surgery, and 52 (10%) acute infection/sepsis. In a multivariable analysis, recent surgery [odds ratio (OR) 2.92, 95% confidence interval (CI) 1.80–4.73] was independently associated with a diagnosis of isolated distal DVT, whereas cancer (OR 2.01, 95% CI 1.20–3.35), male sex aged 41 to 75 years (OR 2.21, 95% CI 1.33–3.67), and acute infection/sepsis (OR 2.71, 95% CI 1.29–5.66) with a diagnosis of proximal DVT. In SWIVTER, age, sex, and several provoking risk factors for VTE appeared to be related with the presenting location of first symptomatic DVT. Cancer, male sex, and acute infection/sepsis were associated with a proximal location of DVT, whereas recent surgery was associated with a distal presentation, likely acting as confounders for the association between thrombosis location and prognosis.

## Introduction

The various clinical manifestations of acute venous thromboembolism (VTE), such as isolated distal (or below-the-knee) deep-vein thrombosis (IDDVT), proximal deep-vein thrombosis (PDVT), or pulmonary embolism (PE) with or without DVT may not share the same pathophysiological mechanisms and provoking factors. This may partly explain prognostic differences [1]. In particular, IDDVT is defined as a thrombus localized below the popliteal veins at the level of axial or calf muscle veins [2] and characterized by a more favorable prognosis, at least in the absence of major persisting risk factors [1].

Age, sex, and their interactions with VTE provoking risk factors may influence the presenting location of a first symptomatic acute DVT [3, 4]. Specifically,  among patients with DVT, adult women, e.g., those aged 40**–**69, had unprovoked IDDVT more frequently than men of the same age, who conversely had unprovoked proximal DVT more often. In both women and men, active cancer is associated with proximal DVT, whereas recent surgery appears to predispose to IDDVT [3].

Based on two recently published analyses investigating impact of demographic and provoking factors on the presenting location of acute DVT [3, 4], we aimed to reproduce these results in the patient population from the SWIss Venous ThromboEmbolism Registry (SWIVTER) and to provide additional insights on risk factors associated with a diagnosis of first isolated distal versus proximal DVT without symptomatic PE.

## Methods

### Patients

Among the 2062 patients enrolled in the SWIss Venous ThromboEmbolism Registry between November 2012 and February 2015, 530 had first symptomatic provoked or unprovoked acute DVT of the lower limbs without concomitant PE. Inclusion criteria were age ≥ 18 years, acute lower-extremity DVT objectively confirmed by diagnostic compression ultrasound or venography, and complete follow-up at 90 days. Both outpatients and inpatients were recruited. Patients with prior thrombosis, incidental VTE, or concomitant symptomatic PE were excluded from the present analysis. The diagnostic workup of acute DVT was managed according to the standard of care at each participating hospital.

The study was approved by local ethics committees of all participating centers and conducted in accordance with the principles of the Declaration of Helsinki.

### Data and definitions

For all enrolled patients, SWIVTER collected individual level data sets. Those data sets included demographics, co-morbidities, and information on DVT presentation, including (i) diagnosis and thrombus location, (ii) risk factors for thrombosis and bleeding, (iii) treatment modalities including reperfusion therapy, type and duration of anticoagulation therapy, and (iv) clinical outcomes at 90 days. De-identified data were entered into a web-based case report form system directly by treating physicians or trained study nurses in each participating hospital.

For the purpose of this post hoc analysis, patients were allocated into two groups according to the most proximal location of deep-vein thrombosis. Isolated distal DVT was defined anatomically as a thrombus located below the popliteal veins at the level of axial or calf muscle veins (IDDVT group). Proximal DVT was defined as any thrombosis located in the popliteal veins or higher up to the right ventricle (PDVT group). The extent of thrombosis was based on evaluation of ultrasound or if available venography reports. Being purely observational, SWIVTER did not require additional imaging studies to define exact thrombus extension or the presence of asymptomatic concomitant PE if not clinically indicated. Patients were classified as having active cancer in the presence of positive clinical finding plus positive imaging or biomarker plus need for any cancer treatment [5]. Non-cancer provoked VTE was defined as thrombosis associated with surgery (incl. major trauma or fracture), estrogen therapy, pregnancy, immobilization for > 3 days (incl. plaster cast), or prolonged (> 8 h) flight, all within 30 days prior to VTE diagnosis [6]. Thrombocytopenia was defined as a platelet count below 100,000/ml.

### Statistical analysis

Normally distributed continuous data were described by mean and standard deviation (SD), and comparisons were performed by use of the *t* test. Continuous data with skewed distribution were depicted as median with interquartile range (IQR), and comparisons were conducted by the use of rank-sum test. Discrete variables were displayed as frequencies with percentages, and comparisons were performed by use of the Chi-square or Fisher’s exact test, where appropriate.

Univariable logistic regression analysis reporting odds ratios (OR) with 95% confidence intervals (CI) was conducted to identify clinical factors associated with initial diagnosis of IDDVT. Subsequently, multivariable logistic regression analysis was performed to identify independent clinical predictors for diagnosis of IDDVT. Univariable factors with a *p* < 0.05 were included in the regression process, and a backward elimination procedure was used to stepwise discard variables without significance from the model.

All reported *p* values are two tailed. Data were analyzed using STATA 13.0 software (STATACorp LP, College Station, Texas, USA).

## Results

Five hundred and thirty patients with a first symptomatic acute DVT without PE were recruited. Mean age was 59 ± 19 years, and 236 (45%) were older than 65. Of these, 184 (35%) patients had IDDVT. Women (*n* = 266) comprised 50% of the overall cohort; 54% of the IDDVT and 48% of the PDVT group. The demographic and baseline characteristics of the study population stratified by DVT localization are displayed in Table 1.

**Table 1:** Demographics, chronic and acute co-morbidities

	Proximal DVT *n* = 346		IDDVT *n* = 184		Total DVT *n* = 530	
Demographics						
Age ≥ 65 years, *n* (%)	142	(41.0)	94	(51.1)	236	(44.5)
Women, *n* (%)	166	(48.0)	100	(54.3)	266	(50.2)
Duration of hospital stay, median days (IQR)	6	(3–12)	12	(9–26)	8	(4–14)
Chronic co-morbidities						
Systemic hypertension, *n* (%)	103	(29.8)	59	(32.1)	162	(30.6)
Cancer, *n* (%)	83	(24.0)	23	(12.5)	106	(20.0)
Hormone replacement, *n* (%)	44	(12.7)	18	(9.8)	62	(11.7)
Severe renal impairment, *n* (%)	35	(10.1)	22	(12.0)	57	(10.8)
Congestive heart failure, *n* (%)	33	(9.5)	22	(12.0)	55	(10.4)
Diabetes mellitus, *n* (%)	29	(8.4)	22	(12.0)	51	(9.6)
Chronic lung disease, *n* (%)	29	(8.4)	14	(7.6)	43	(8.1)
History of stroke/TIA, *n* (%)	13	(3.8)	7	(3.8)	20	(3.8)
Hepatic impairment, *n* (%)	9	(2.6)	4	(2.2)	13	(2.5)
Acute co-morbidities within 30 days						
Prior hospitalization, *n* (%)	47	(13.6)	55	(29.9)	102	(19.2)
Bed rest > 3 days, *n* (%)	53	(15.3)	38	(20.7)	91	(17.2)
Surgery, *n* (%)	37	(10.7)	49	(26.6)	86	(16.2)
Acute infection/sepsis, *n* (%)	42	(12.1)	10	(5.4)	52	(9.8)
Acute inflammatory/rheumatic disease, *n* (%)	17	(4.9)	10	(5.4)	27	(5.1)
ICU admission, *n* (%)	10	(2.9)	15	(8.2)	25	(4.7)
Bleeding at the time of VTE diagnosis, *n* (%)	9	(2.6)	10	(5.4)	19	(3.6)
Pregnancy or postpartum period, *n* (%)	10	(2.9)	4	(2.2)	14	(2.6)
Thrombocytopenia, *n* (%)	10	(2.9)	3	(1.6)	13	(2.5)
Ischemic stroke or palsy, *n* (%)	3	(0.9)	9	(4.9)	12	(2.3)
Acute respiratory failure, *n* (%)	7	(2.0)	3	(1.6)	10	(1.9)
Acute heart failure, *n* (%)	6	(1.7)	4	(2.2)	10	(1.9)
Acute coronary syndrome, *n* (%)	2	(0.6)	3	(1.6)	5	(0.9)

*DVT* deep vein thrombosis, *ID* isolated distal, *IQR* interquartile range, *TIA* transient ischemic attack, *ICU* intensive care unit, *VTE* venous thromboembolism

Among patients aged 41 to 75 years, IDDVT was more frequently diagnosed in women than in men (44% vs. 31%; *p* = 0.017), whereas the opposite was true for PDVT. This sex-specific difference in the proportion of IDDVT was minimal in patients between 18 and 41 years of age (29% in women vs. 36% in men) and above 75 years (35% in women vs. 34% in men).

For the subsequent analysis, we adapted the age groups based on prior data on age-specific distributions of provoking risk factors, notably surgery. In patients with provoked non-cancer associated DVT, a higher proportion of IDDVT was present in women than in men aged 55**–**75 years (53% vs. 43%) and in men than in women between 18 and 54 years (55% vs. 35%). In patients with unprovoked DVT, a higher proportion of IDDVT was present in women than in men aged 55**–**75 years (42% vs. 24%) as well as between 18 and 54 years (30% vs. 22%).

In a multivariable analysis performed in the overall population, recent surgery was associated with a diagnosis of IDDVT, whereas cancer, men aged 41–75 years, and acute infection/sepsis with a diagnosis of proximal DVT (Table 2a and b). Among patients with unprovoked DVT, both age ≥ 65 years (OR 1.67, 95% CI 1.03–2.71; *p* = 0.037) and female sex (OR 1.65, 95% CI 1.03–2.66; *p* = 0.039) were independently associated with a diagnosis of IDDVT.

**Table 2:** (a) Clinical factors associated with the diagnosis of IDDVT, (b) clinical factors associated with the diagnosis of proximal DVT

Analysis factor	Univariable			Multivariable		
	OR	95% CI	*p*	OR	95% CI	*p*
(a)						
Surgery	3.03	1.89–4.86	< 0.001	2.92	1.80–4.73	< 0.001
(b)						
Acute infection/sepsis	2.40	1.18–4.91	0.016	2.71	1.29–5.66	0.008
Men aged 41–75 years	1.76	1.10–2.81	0.018	2.21	1.33–3.67	0.002
Cancer	2.21	1.34–3.65	0.002	2.01	1.20–3.35	0.008

*IDDVT* isolated distal deep-vein thrombosis, *OR* odds ratio, *CI* confidence interval, *DVT* deep-vein thrombosis

## Discussion

In this analysis of the SWIss Venous ThromboEmbolism Registry, we have reproduced prior findings from the RIETE registry (and other cohort studies) in a smaller cohort of patients enrolled in tertiary and secondary centers across Switzerland [3]. We confirmed that age, sex, and several provoking risk factors for VTE influence the presenting location of first symptomatic acute DVT without concomitant PE. Despite an overall equal distribution of IDDVT between sexes, women aged between 41 and 75 years more frequently presented with distal DVT compared with men of the same age group. Our results also emphasize that some of the provoking risk factors for VTE, such as recent surgery was associated with a distal presentation, whereas others, namely cancer, led to an increased risk of presenting with proximal DVT. The specific distribution of these provoking factors, which we showed varying between sexes and across age groups, partly explains the different proportion of IDDVT in women and men.

In our study, approximately one-third of the patients with first DVT had an IDDVT. This observation is consistent with prior studies, although some heterogeneity has been described, also reflecting diagnostic uncertainties and lack of protocols for the diagnosis of isolated distal DVT [3, 7, 8]. We cannot exclude that different strategies for the identification of IDDVT have been adopted at the participating centers. However, given that SWIVTER is a national registry performed at centers referring to the same national guidelines and recommendations for DVT diagnosis, it is likely that the heterogeneity is smaller than in multinational studies [9].

Recent surgery was associated with the isolated distal presentation of DVT as in prior studies [3, 4, 9]. This is likely to be due to surgery-related prolonged immobilization of lower limbs, local pain, and uncertainty/anxiety to mobilize. Alternatively, the observed difference may be simply due to more (or earlier) diagnostic tests performed in this patient group. Vice versa, patients with cancer and acute infection/sepsis had more often proximal thrombosis, possibly reflecting a higher thrombus burden or the “descending” (toward distal) nature of these thrombi, in contrast to an “ascending” propagation (toward proximal) in other patients. The latter includes, for instance, pregnancy, which has been shown in prior studies [3] to be associated with a proximal location of DVT, likely due to local compression of the iliac veins. As for other venous symptoms (especially varicose vein symptoms), women might tend to see a physician earlier than men, and in line with the ascending thrombus extension theory, this might partly explain the more frequent diagnosis of IDDVT in women. In SWIVTER, the prevalence of pregnancy was low and we could not confirm the association with a proximal location of DVT which has been shown in prior studies [3], likely due to local compression of the iliac veins.

Our analysis has some limitations. First, as IDDVT does not consist of a unique phenotype and may encompass thrombosis in both the axial and muscular veins, the latter possibly being subject to physician judgment owing to the lack of an established diagnostic guidance, we cannot exclude differences between centers in the diagnosis assignment. Second, the numbers of patients with IDDVT was moderate and consequently, the results of multivariable analysis should be interpreted with caution. Third, we did not include patients with PE and based our analysis on the diagnosis of DVT guided by the presenting symptoms, similarly to the work of Barco et al. [3]. Finally, SWIVTER was conducted in the Swiss centers only and the results may not be generalizable to other countries or territories characterized by different healthcare systems.

## Conclusions

We confirmed that age, sex, and several provoking risk factors for VTE were related to the presenting location of first symptomatic DVT without concomitant PE. Cancer, male sex, and acute infection/sepsis were associated with a proximal location of DVT, therefore, acting as confounders for the association between thrombosis location and recurrence. One cannot exclude that other factors act as unaccounted confounders for the association between demographic factors and DVT location, and between DVT location and outcomes.
